# Changes in habitat associations during range expansion: disentangling the effects of climate and residence time

**DOI:** 10.1007/s10530-017-1616-9

**Published:** 2017-11-22

**Authors:** Martin J. P. Sullivan, Aldina M. A. Franco

**Affiliations:** 1 School of Environmental Sciences, University of East Anglia, Norwich Research Park, Norwich, NR4 7TJ UK; 2 School of Geography, University of Leeds, Leeds, LS2 9JT UK

**Keywords:** Range expansion, Density-dependent habitat use, Species distribution modelling, Species–environment relationship, Common waxbill

## Abstract

The distributions of many species are not at equilibrium with their environment. This includes spreading non-native species and species undergoing range shifts in response to climate change. The habitat associations of these species may change during range expansion as less favourable climatic conditions at expanding range margins constrain species to use only the most favourable habitats, violating the species distribution model assumption of stationarity. Alternatively, changes in habitat associations could result from density-dependent habitat selection; at range margins, population densities are initially low so species can exhibit density-independent selection of the most favourable habitats, while in the range core, where population densities are higher, species spread into less favourable habitat. We investigate if the habitat preferences of the non-native common waxbill *Estrilda astrild* changed as they spread in three directions (north, east and south-east) in the Iberian Peninsula. There are different degrees of climatic suitability and colonization speed across range expansion axes, allowing us to separate the effects of climate from residence time. In contrast to previous studies we find a stronger effect of residence time than climate in influencing the prevalence of common waxbills. As well as a strong additive effect of residence time, there were some changes in habitat associations, which were consistent with density-dependent habitat selection. The combination of broader habitat associations and higher prevalence in areas that have been colonised for longer means that species distribution models constructed early in the invasion process are likely to underestimate species’ potential distribution.

**Electronic supplementary material:**

The online version of this article (10.1007/s10530-017-1616-9) contains supplementary material, which is available to authorized users.

## Introduction

The distributions of many species are not static. Species are shifting their ranges in response to climate change (Gillings et al. 2015; Hickling et al. 2006; Hill et al. 1999; Parmesan and Yohe 2003), while species transported to new areas by humans are spreading to suitable areas in their non-native range (Sullivan et al. 2012; Václavík and Meentemeyer 2012). Species distribution models are commonly used to predict the potential distribution of these species (Early and Sax 2014; Jimenez-Valverde et al. 2011; Peterson 2003). For example, the environmental associations of a non-native species can be characterised using their native distribution and/or current distribution in their non-native range (Broennimann and Guisan 2008; Mau-Crimmins et al. 2006), and used to identify other areas which share these suitable environmental conditions and so could potentially be colonised in the future (Fischer et al. 2016; Jimenez-Valverde et al. 2011). This approach typically assumes spatial and temporal stationarity in species’ environmental associations. This assumption may be violated, as species sometimes show greater habitat specificity at expanding range margins (Oliver et al. 2009), while increasing temperatures can increase niche breadth and allow species to exploit new resources during range expansion (Pateman et al. 2012) or interact with microclimate to cause shifts in species habitat associations (Davies et al. 2006). Furthermore, many non-native species, across a range of taxa, appear to show niche shifts between their native and non-native range (Broennimann et al. 2007; Cornuault et al. 2015; Stiels et al. 2015), although there is debate as to the extent these niche shifts are biologically meaningful (Petitpierre et al. 2012; Strubbe and Matthysen 2014). Additionally, most studies focus on climate niche rather than other aspects of species’ niche (Larson et al. 2010), such as habitat association.

Understanding if changes in habitat preferences occur during range expansion will be important to evaluate whether the assumption of stationarity is justified in species distribution models of non-native species. If changes in habitat preference are common, techniques such as geographically weighted regression can be used to explore and account for non-stationarity (Osborne et al. 2007), but these do not capture the mechanisms that lead to non-stationarity. Therefore, it is also be important to understand why habitat preferences change in order to inform attempts to incorporate non-stationarity in habitat preferences into species distribution models.

Variation in habitat associations between areas that have been colonised for a long time (the range core) and areas that have been recently colonised (the range margin) may be driven by climate. For example, butterfly species in the UK have been found to exhibit higher habitat specificity as they spread into areas with less favourable climate (Oliver et al. 2009), while for endothermic species, climate and habitat may interact as resource rich habitats can enhance survival and breeding success in unfavourable climates (Robb et al. 2008). Alternatively, lower population densities in range margins may lead to differences in habitat associations if species exhibit density dependent habitat selection (Brown 1984), where the most favourable habitats are occupied at low population densities, in the early colonisation stage, and less favourable habitats are only occupied once the more favourable habitats become saturated as population density increases (Morris 1987; Sullivan et al. 2015b). If this was occurring, species would be expected to occupy a wider range of habitats in areas that have been colonised for a long time, and hence population densities are higher, than in recently colonised areas.

Disentangling the role of climate and residence time in influencing the habitat associations of range expanding species is challenging as they are often confounded, with range expanding species moving into climatically marginal areas. The spread of non-native species provides an opportunity to disentangle the effects of climate and residence time, as species are not necessarily moving into less suitable climates in all expansion axes, hence recently colonised areas will have varying climatic suitability. The expansion of the common waxbill *Estrilda astrild* in the Iberian Peninsula provides such an opportunity. We assess the importance of climate and residence time in influencing the habitat associations of common waxbills. Our aims are to (1) quantify the habitat associations of common waxbills, (2) test whether these vary with residence time or with climate and (3) evaluate the importance of residence time and climate in influencing patterns of occurrence.

## Methods

We employ a space-for-time substitution to test whether the habitat associations of common waxbills vary with residence time or climate as they expand their range. Focal watches were carried out to identify habitat features that are important for common waxbills. We then modelled the occurrence of common waxbills in 349 point counts as a function of habitat features identified to be important by the focal watches, as well as climate and residence time.

### Field survey

We sampled along three main directions of common waxbill range expansion in their European non-native range. These expansion axes were along the west coast of Portugal from introduction sites near Lisbon and Óbidos, along the south coast of Portugal into south-west Spain from introduction sites in the Algarve, and along the Guadiana Valley east into Spain (Silva et al. 2002). This sampling design enabled the influence of residence time to be disentangled from climate, as climate conditions varied between expansion axes. For example, common waxbills introduced to the Lisbon area spread along the west coast of Portugal through areas identified to be climatically suitable by Sullivan et al. (2012), and also eastwards into less climatically suitable areas such as Extremadura.

**Fig. 1:**
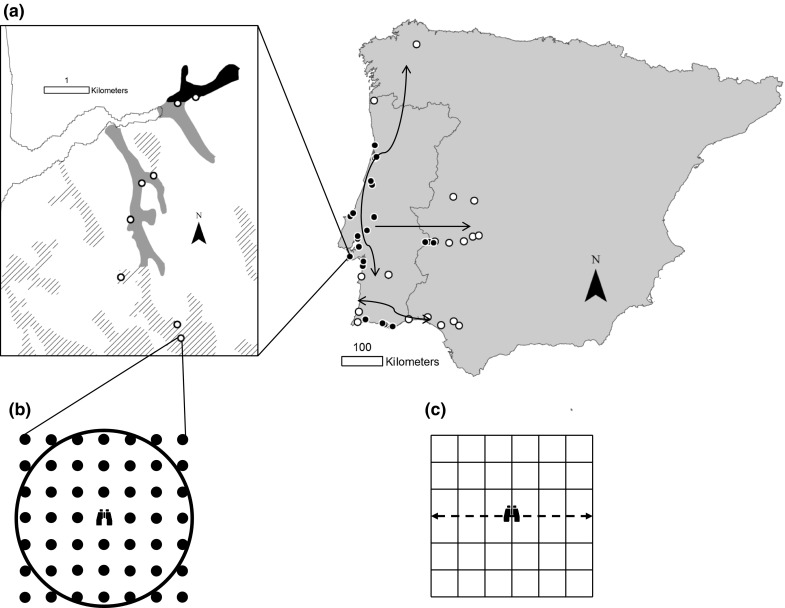
**a** Location of survey sites in the Iberian Peninsula. The centroids of each site are plotted. Sites colonised before 1990 are shown by filled circles, and colonised after 1990 are shown by open circles. Arrows show axes of range expansion. The insert map shows the location of point counts at one site. Point count locations are shown by open circles. Rice fields are shaded grey, wetlands shaded black, and heterogeneous agriculture (Corine land-cover level two class 24) shown by hashing. The remaining area is largely forestry. **b** Schematic of sampling protocol at each point count. The observer (position shown by binoculars) records birds seen within a 100 m radius (shown by circle). Habitat is recorded at regularly spaced points (shown by filled circles, habitat also recorded at position of observer). **c** Schematic of sampling protocol at focal watch locations. The observer walks along a central transect (dashed arrow), and records birds and percentage cover of habitats in each sub-square

We selected 41 10 × 10 km UTM squares (referred to as sites) that contained potentially suitable habitat for common waxbills (Reino and Silva 1998; Sullivan et al. 2012). These potentially suitable habitats were rice fields and irrigated agriculture (Corine land-cover (CLC) classes 212 and 213), wetlands and rivers (CLC 411 and 511), and heterogeneous agriculture (CLC level two class 24). At each site, five to 12 point counts (mean = 8.5 ± 2.5 SD point counts per site) were carried out in these habitats, with the number of point counts varying depending on the extent of accessible suitable habitat. These point counts were located in or around the selected 10 km square (see Fig. 1 for locations of site centroids). In total 349 point counts were performed. Point counts were always > 200 m apart. Sites could be located in adjacent 10 km squares, but point counts in each site were non-overlapping. Sites were assigned a residence time based on the date the 20 km × 20 km UTM grid-cell their centroid fell in was colonised, using colonisation data from Silva et al. (2002). The dataset compiled by Silva et al. (2002) combined published records of common waxbills with further records from correspondence with birdwatchers in Portugal and Spain to obtain the earliest record in each 20 × 20 km UTM square (Reino 2005; Reino et al. 2009; Reino and Silva 1998). We selected sites to provide an approximately balanced sampling design by residence time (< 10 years, n = 8; 10–20 years, n = 10; 20–30 years, n = 10; > 30 years, n = 13), and ensure the full ranges of residence times in each expansion axis were sampled. There were at least 20 point counts in each habitat class in each residence time strata (Table 1). Seasonal effects were controlled for by surveying each expansion axis three times during the fieldwork period (April–June 2011), surveying a third of sites in each residence time strata in each period, as well as by including survey date as a covariate in subsequent statistical models.

**Table 1:** Proportion of point counts in each habitat and residence time strata where common waxbills were recorded

Residence time (years)	Irrigated agriculture (CLC 212, 213)	Wetland (CLC 411, 511)	Heterogeneous agriculture (CLC 24)	Total
> 30	19/39 (49%)	10/40 (25%)	13/27 (48%)	42/106 (40%)
20–30	13/23 (57%)	15/28 (54%)	12/22 (55%)	40/73 (55%)
10–20	9/31 (29%)	12/30 (40%)	10/30 (33%)	31/91 (34%)
< 10	5/21 (24%)	8/36 (22%)	4/22 (18%)	17/79 (22%)
Total	46/114 (40%)	45/134 (34%)	39/101 (39%)	130/349 (37%)

Data are presented as number of point counts where common waxbills were present/total number of point counts, with the percentage of point counts where common waxbills were present in parenthesis

At each point count location, the presence or absence of common waxbills during a 5 min point count was recorded, with the distance from observer and flock size of each individual or group of common waxbills also noted. Flock size was noted as we expected flocks to be easier to detect than individuals as birds in flocks make contact calls. The habitat classes present (see Table 2 for habitat classes) at 30 m intervals on a grid stretching 90 m in each cardinal direction from the point count location were recorded (i.e. 49 habitat recording points per point count, see Fig. 1b for schematic). The presence or absence of a river within 100 m of the point count location was noted. This scale enabled the majority of common waxbills to be detected, and therefore represented the resources that directly influenced the occurrence of common waxbills at sampling points. All point counts, including assessment of available habitat, were performed by the same observer (MS).

**Table 2:** Microhabitat selection by common waxbills, calculated using Jacobs index (*J*)

Habitat type	Feeding		Shelter	
	*N*	*J*	*N*	*J*
Rough grass	34	0.35*	6	− 0.70*
Emergent vegetation	19	0.45*	44	0.75*
Forbs	18	0.14	13	− 0.07
Houses and gardens	1	− 0.23	1	− 0.43
*Arundo donax*	3	− 0.03	12	0.77*
Trees and bushes	5	− 0.58*	17	0.10
Crops	6	− 0.63*	3	− 0.84*

*N* is the number of observations of each activity in each habitat. Asterisks indicate that microhabitat use differs statistically significantly from expected use if each microhabitat was selected randomly (assessed by expected use of a microhabitat falling outside the 95% Bonferoni confidence intervals of observed proportional use). In total there were 96 observations of feeding and 98 observations of shelter; in addition to observations included in this table, ten observations were of ground feeding birds where it was not certain which microhabitat was being used, while two shelter observations were of birds perched on bare ground. Data were obtained from focal watches at 68 locations, with feeding and shelter activities of common waxbills observed at 27 and 26 locations respectively

### Climate data

We selected two climate variables that we expected to influence common waxbill occurrence and potential habitat associations: mean temperature in the coldest month (MTCM) and cumulative water deficit (CWD). MTCM could affect habitat associations as birds require more energy to survive colder winters (Newton 1998) so they may be restricted to habitats that provide more resources. The effect of MTCM on breeding habitat associations may be reduced by movements between the breeding and non-breeding seasons, however as common waxbills are largely sedentary, limits on winter habitat associations are likely to carry over to affect breeding habitat associations. MTCM was extracted from the Worldclim database (Hijmans et al. 2005) from the 1 km grid-cell containing each point count. CWD was calculated by first calculating the water deficit in a given month as the difference between monthly precipitation and monthly evapotranspiration, plus cumulative water deficit in the previous month. We then took the minimum value of cumulative water deficit reached over the year. Values of CWD were obtained from a database compiled by Chave et al. (2014). CWD reflects the degree of drought stress an area experiences. Common waxbills may be more associated with wetland habitat features (rivers and emergent vegetation) in areas experiencing greater drought stress (Barnard 1997). We also examined whether habitat associations varied with a multivariate assessment of climate suitability by using the predicted suitability from a dispersal weighted species distribution model (suitability values taken from Sullivan et al. 2012). This used generalised linear models to relate the occurrence of common waxbills in 10 km grid cells in the Iberian Peninsula to MAT, mean annual precipitation and mean daily temperature range (see Sullivan et al. 2012 for a full description of this model). We call this variable Climate SDM. Residence time was weakly correlated with CWD (*r* = 0.33), with stronger correlations with MAT (*r* = 0.55) and Climate SDM (*r* = 0.72). Habitat variables were weakly correlated with climate and residence time (|*r|* ≤ 0.31); this variation in habitat prevalence is implicitly accounted for in our subsequent analysis by using presence-absence models (see “Data analysis”) which consider the prevalence of different habitats in point counts where common waxbills are present and absent.

### Quantifying resource selection

We investigated how common waxbills use different habitat features for feeding and shelter to identify habitat features that provide important resources. This microhabitat selection was quantified by performing scan samples at 68 locations located throughout residence time strata. Habitat availability was recorded in a 180 m × 180 m square, divided into 30 m × 30 m sub-squares. The percentage cover of each habitat type was recorded in each sub-square. By recording the amount of habitat in sub-squares at different distances from the observers we were able to adjust the calculation of habitat availability to account for the decline in detectability with distance from observer (see Appendix S1 for details and Fig. 1c for schematic). Habitat use by common waxbills was recorded in scan samples performed every 10 min, with the observer allowed to walk up and down a transect crossing the middle of the recording area. During each scan sample the distance from observer, habitat use and activity (feeding or shelter) of each group of common waxbill was recorded. Shelter was defined as any rest activities while not feeding. We quantified the selection of each habitat, given availability, for each activity using Jacobs index (Jacobs 1974), where Jacobs index for habitat *h* and activity *a* is *J*_*h,a*_ = (*O*_*h,a*_ − *E*_*h,a*_)/(*O*_*h,a*_ + *E*_*h,a*_ − 2*O*_*h,a*_ *E*_*h,a*_), where *O*_h,a_ is the number of observations of activity *a* in habitat *h*, and *E*_*h,a*_ is the expected number of observations if the habitat was selected in proportion to its availability (see Appendix S1 for further details). Jacobs index ranges between − 1 and 1, and equals zero if a habitat is selected in proportion to its availability, is positive if a habitat is selected more than expected given availability, and is negative if a habitat is selected less than expected given availability.

### Data analysis

We follow a two-step approach to modelling the occurrence of common waxbills (Miller et al. 2013) where we first use distance sampling to model the detection probability of common waxbills at each point count location, then use the predicted detection probabilities as an offset in models of common waxbill occurrence to account for spatial heterogeneity in detectability (Massimino et al. 2015).

We constructed models of the probability of detecting common waxbills, with gamma functions modelling the decline in detection probability with distance from the observer, using the R package mrds (Laake et al. 2015). Gamma functions were selected as they resulted in models with lower AIC than when half-normal, hazard-rate or uniform functions were used. The quantity of emergent vegetation and trees and bushes were included as covariates (these were quantified as the proportion of habitat recording points that contained these habitat features), as these tall habitat features could obscure birds. Flock size was included as a covariate, as larger flocks may be easier to detect as they make more contact calls. We fitted all simplifications of this model, and used AIC to rank models (Table S1). The best performing model (with flock size and amount of trees and bushes as covariates) was used to estimate the detection probability in each point count location, but set flock size to one when making predictions so that variation in modelled detection probability is only based on variation in habitat.

We then modelled the presence/absence of common waxbills at point count locations using generalised linear mixed effects models with binomial errors and a logit link. We formulated competing hypotheses to explain variation in the occurrence of common waxbills, and constructed models that represented these hypotheses (Table 3). These models range in complexity from a null model without any habitat terms, through to models with only habitat terms (assuming that climate or residence time do not affect fine-scale occurrence), models with an additive effect of climate or habitat (assuming that habitat associations do not vary with climate or residence time, but climate or residence time affects the prevalence if common waxbills) and finally to models with interaction terms which allow habitat associations to vary with residence time or with climate (Table 3). We selected habitat variables for inclusion in these models based on their use for feeding and shelter as indicated by positive Jacobs index values (Table 2), meaning that models contained terms relevant to resource availability. These were emergent vegetation (including *Arundo donax*), trees and bushes, forbs, and rough grass The presence of a river within 100 m of the point count location was also included as a habitat variable as common waxbills have been reported to be associated with riverine vegetation (Reino and Silva 1998). Habitat variables (except for the presence of a river, which was a binary factor), residence time and climate were modelled using second order polynomial terms to allow for non-linear relationships. Where models contained interaction terms with habitat variables, these were with both first and second order terms. We used AIC to evaluate the relative support for each model as it allows comparison of models that are not nested (Burnham and Anderson 2002). Models were constructed in a mixed effects framework, with a random intercept site effect to account for the expected correlation of observations within each site (this was sufficient to account for residual spatial autocorrelation, Fig. S1), using the R package lme4 (Bates et al. 2014). All models contained a survey date term to model seasonal variation in occurrence that could occur due to the swelling of common waxbill populations by fledglings later in the season, as well as the logit of the predicted detection probability of each point count location as an offset to account for variation in detectability. The explanatory power of the fixed effects component of these models was quantified by calculating the marginal *R* ^2^ (Nakagawa and Schielzeth 2013).

**Table 3:** Hypotheses to explain variation in the occurrence of common waxbills, and corresponding statistical models

Hypothesis	Model explanatory variables
1. Occurrence related to the extent of habitat used for feeding and shelter. These habitat associations remain constant throughout the range	Detect + Date + Habitat
2. Occurrence related to habitat and residence time. Habitat associations remain constant throughout the range	Detect + Date + Habitat + Residence time
3. Occurrence related to habitat and climate. Habitat associations remain constant throughout the range	Detect + Date + Habitat + CWD
	Detect + Date + Habitat + MTCM
	Detect + Date + Habitat + Climate SDM
4. Occurrence related to habitat and residence time. Habitat associations vary with residence time	Detect + Date + Habitat * Residence time
5. Occurrence related to habitat and climate. Habitat associations vary with climate	Detect + Date + Habitat * CWD Detect + Date + Habitat * MTCM Detect + Date + Habitat * Climate SDM
6. Occurrence not related to habitat, residence time or climate	Detect + Date

Interactions between variables are shown by *. Habitat variables are forbs, rough grass, emergent vegetation and trees and bushes, all expressed as the proportion of habitat recording points containing these habitat classes, and the presence of a river. Second order polynomial terms were included for continuous habitat variables, climate and residence time. Detect is the logit detection probability at a point count location, and is included in models as an offset

## Results

### Habitat associations of common waxbills

Common waxbills selected rough grass, emergent vegetation and forbs for feeding (Table 2). Emergent vegetation and *A. donax* were strongly selected for shelter, with weaker selection for trees and bushes (Table 2). In locations colonised for less than 20 years, forbs and trees and bushes were not selected for feeding and shelter more than expected given availability (Table S2), but in general there were too few observations of feeding or shelter to robustly test whether microhabitat selection varied during range expansion.

**Fig. 2:**
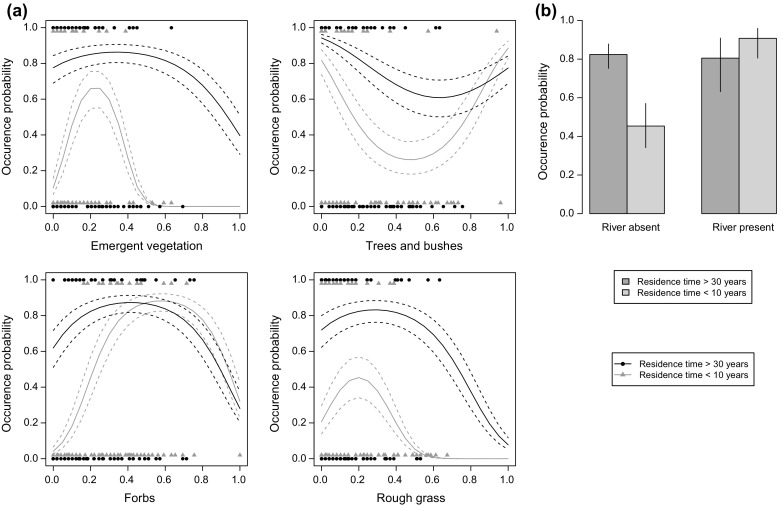
Interactions between habitat and residence time in explaining the occurrence of common waxbills. **a** Relationships between occurrence probability and the proportion of habitat recording points containing each variable. Relationships have been shown for the oldest residence time strata (areas colonised before 1980, black) and the most recent residence time strata (areas colonised after 2000, grey) to visualise the effect of residence time on habitat associations. Dashed lines show 95% confidence intervals around relationships. **b** Occurrence probability at point counts where rivers are present or absent in areas colonised before 1980 (dark grey) and after 2000 (light grey). Error bars show 95% confidence intervals. Both (**a**) and (**b**) are based on predictions from model 4 in Table 3 holding other variables at their overall mean; note that this means occurrence probabilities are generally high as these other variables have values close to their optimum. *N* = 349 point counts in 41 sites

Common waxbills were recorded in 130 of the 349 point counts. The probability of recording common waxbills did not differ significantly between the three aggregated CLC habitat classes sampled (likelihood ratio test with nested model lacking habitat class term, $\chi_{\,\,\,2}^{2} = 0.26$, *P* = 0.88, Table 1), however differences in habitat suitability were evident within these broad habitat classes. Relationships between common waxbill occurrence and the amount of emergent vegetation, forbs and rough grass were humped, indicating a preference for intermediate values of these habitat features. The relationship with the amount of trees and bushes was negative over the range of tree and bush extent where we have most data, indicating that higher coverage of trees and bushes was avoided (Fig. 2). This relationship switched to being positive when > 50% of habitat sampling points contained trees and bushes, which could indicate selection of areas with high tree cover for shelter, but as this switch from negative was driven by the occurrence of common waxbills at a few point counts with high tree/bush cover it is unlikely to be robust.

### Effect of climate and residence time

**Fig. 3:**
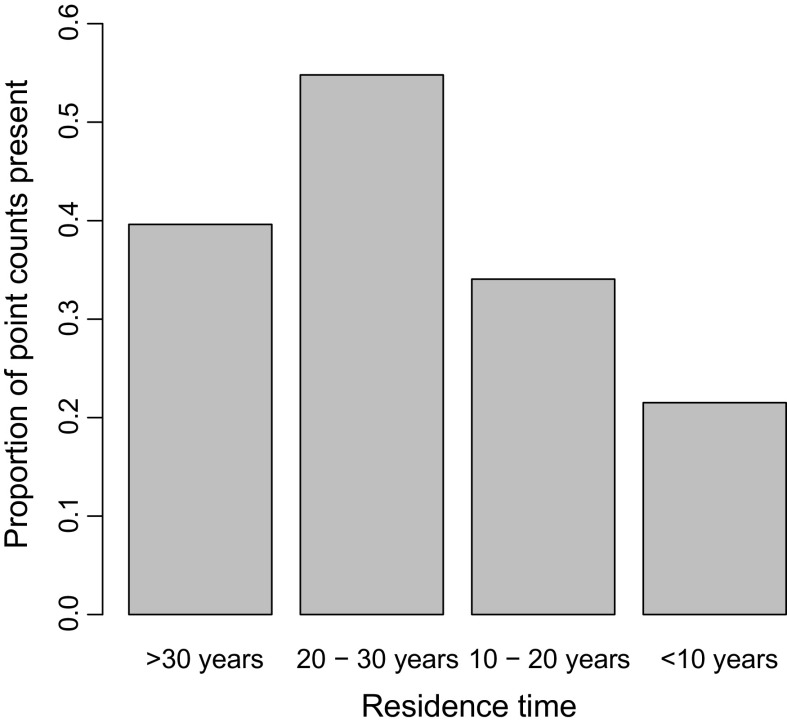
Proportion of count counts where common waxbills were recorded in each residence time strata. *N* = 349 point counts in 41 sites

Residence time was supported as a predictor variable, appearing in the two best supported models (Table 4). The probability of a point count being occupied increased with residence time, peaking at sites that had been colonised for at least 20 years (Fig. 3). There was some uncertainty over whether habitat associations changed with residence time; despite a substantial increase in model explanatory power by having interactions between habitat variables and residence time, improvements to AIC were small (ΔAIC = 2.6) due to the associated increase in model complexity (Table 4). The most marked change in habitat preferences was a tolerance of a wide range of emergent vegetation cover in areas colonised for over 30 years, contrasting with a preference for intermediate amounts of emergent vegetation in areas that has been colonised for no more than 10 years (Fig. 2). The presence of a river also had a positive effect on occurrence in areas colonised within 10 years, but was not important in areas colonised for over 30 years (Fig. 2). Increased residence time lead to greater tolerance to areas with fewer forbs and more rough grass (Fig. 2). Despite these changes in fine-scale habitat associations, the proportion of occurrences in the three habitat classes (irrigated agriculture, heterogeneous agriculture and wetlands) did not change with residence time (likelihood ratio test between models with and without habitat class: residence time interaction term, $\chi_{\,\,\,6}^{2} = 7.5$, *P* = 0.28, Table 1).

**Table 4:** Performance of models explaining patterns of common waxbill occurrence

Model	Log Likelihood	Parameters	ΔAIC	Marginal *R* ^2^
Detect + Date + Habitat * Residence time	− 175.1	32	0	0.544
Detect + Date + Habitat + Residence time	− 194.4	14	2.5	0.315
Detect + Date + Habitat + Climate SDM	− 197.4	14	8.6	0.278
Detect + Date + Habitat + MTCM	− 197.6	14	9.0	0.281
Detect + Date + Habitat	− 200.4	12	10.5	0.244
Detect + Date + Habitat + CWD	− 198.4	14	10.5	0.274
Detect + Date + Habitat * MTCM	− 185.8	32	21.3	0.435
Detect + Date + Habitat * Climate SDM	− 187.4	32	24.5	0.363
Detect + Date + Habitat * CWD	− 191.2	32	32.1	0.493
Detect + Date	− 222.4	3	36.6	0.066

Interactions between variables are shown by *. Habitat variables are forbs, rough grass, emergent vegetation and trees and bushes, all expressed as the proportion of habitat recording points containing these habitat classes, as well as the presence or absence of a river. Logit detection probability was incorporated in models as an offset. *N* = 349 point counts in 41 sites

Climate was poorly supported as an additive effect (Climate SDM ΔAIC from best model = 8.7, MTCM ΔAIC = 9.1, CWD ΔAIC = 10.6), with less support for interactions between climate and habitat variables (Table 3). The null model, containing only season and detection probability as fixed effects, was the least supported model (ΔAIC = 34.5, Table 4).

## Discussion

Residence time had more support than climate suitability in influencing variation in the fine-scale prevalence of common waxbills across their European non-native range. There was support for interactions between habitat variables and residence time. In the early stages of colonisation, common waxbills are strongly associated with rivers and areas with intermediate amounts of emergent vegetation (Fig. 2). This association with rivers suggests these landscape features have a role in assisting dispersal, as they provide corridors of suitable habitat that facilitate common waxbill dispersal. Previous studies have documented the role of dispersal along linear landscape features, such as rivers, in facilitating the spread of non-native species at expanding range margins (e.g. Brown et al. 2006). This could lead to anisotropic range expansion (Hengeveld 1989), which would need to be accounted for in models of species’ range expansion (Fitzpatrick et al. 2012). Alternatively, rivers and their associated riparian vegetation may also be important in recently colonised areas due to density dependent habitat selection (Brown 1984; Morris 1987), with rivers being preferred habitats that are occupied when populations are at low densities, and areas away from rivers less preferred so only occupied at higher population densities. Residence time influences the relationships between common waxbill occurrence and emergent vegetation in a way that is consistent with the occurrence of buffer effects, observed by the more restricted habitat associations at expanding range margins. The positive unimodal relationship between common waxbill occurrence and the quantity of emergent vegetation was most pronounced in recently colonised areas, where common waxbills were most likely to be recorded at point count locations containing 20% emergent vegetation. In areas occupied for longer, common waxbills were likely to occur across a wide gradient of emergent vegetation quantity. These changes in habitat association revealed by our space-for-time substitution are consistent with anecdotal reports that common waxbills introduced to Portugal were initially restricted to wetland edges before spreading to a wider range of habitats (Reino and Silva 1998). Despite these changes in preference for local habitat features, they do not appear to be strong enough to affect coarser scale habitat associations, as we did not detect any shift in association with land-cover classes with residence time. Thus, species distribution models relating to occurrence to land-cover (e.g. Fischer et al. 2016) are unlikely to have been affected by the variation in habitat preference documented here.

Our results also show that the additive effect of residence time was a strong influence on local occurrence of common waxbills. This effect was independent of measured habitat variables, meaning that common waxbills are less likely to occur at range margins regardless of habitat, although it is possible that habitat varied between the range core and range margin in ways that were not captured in this study. The prevalence of common waxbills took approximately 20 years to saturate following colonisation of an area, supporting previous work reporting long lag phases in biological invasions (Shigesada et al. 1995; Wangen and Webster 2006). These lags have two important consequences. Firstly, the lower population densities in the early stages of invasion allow native and non-native species to coexist through mechanisms which may not be stable when non-native species reach higher population densities (Grundy et al. 2014; Newson et al. 2011), complicating the early assessment of non-native species’ impacts. Secondly, the lower prevalence of recently established non-native species means that species distribution models trained on these early distributions are likely to underestimate the potential distribution of these species, even if environmental associations are shown to be consistent with the assumption of stationarity. Our results indicate that this will be a particular problem when fine-scale distribution data is used, as common waxbills were able to spread to new areas before reaching equilibrium prevalence within colonised areas.

Our results contrast with previous studies that have documented an effect of climate on habitat associations in expanding range margins (Lawson et al. 2014; Oliver et al. 2009). The absence of a strong effect of climate could be because this study looked at an endotherm, while previous studies that have found strong climate-habitat interactions have looked at ectotherms (Lawson et al. 2014; Oliver et al. 2009) where interactions were partially driven by the microclimates provided by different habitats (Suggitt et al. 2012); habitat is unlikely to modulate the physiological effects of climate to the same extent in endotherms. Despite this, climate could plausibly interact with the common waxbill’s habitat associations in several ways. Firstly, winter survival is related to a bird’s energy balance; in order to survive cold weather birds need to increase their food intake (Newton 1998; Siriwardena et al. 2008), so common waxbills may be restricted to higher quality habitats in colder areas (this could influence breeding habitat associations as common waxbills are not migratory). Secondly, common waxbills typically breed in mesic habitats (Reino and Silva 1998), and in areas of their native range with arid climates they are restricted to wetlands (Barnard 1997). We did not find support for such interactions, which may indicate that climatic conditions in the range margin are not sufficiently harsh to affect habitat associations. Residence time effects are likely to be more pronounced in birds than invertebrates, as population densities of the latter can increase rapidly at range margins (Bourn and Thomas 2002), potentially reducing differences in population density with residence time.

### Habitat associations of common waxbills

Common waxbills strongly selected emergent vegetation for shelter and moderately selected forbs, rough grass and emergent vegetation for feeding. Similar patterns of resource selection are evident in other non-native seed-eating birds in the Iberian Peninsula (Sullivan et al. 2015a). The presence of these resources influenced occurrence, with humped shaped relationships with these variables indicating that common waxbills were associated with areas with intermediate amounts of these resources. Models containing habitat variables had substantially greater explanatory power than the null model only containing date and detection probability (Table 4), and a model containing habitat but not residence time had more support than a model containing residence time but not habitat (ΔAIC = 21.0), supporting the role of these habitat variables in influencing patterns of occurrence. The association of common waxbills with emergent vegetation reflects habitat associations in their native range, where although common waxbills are associated with a wide range of habitats they are particularly strongly associated with wetland vegetation (Barnard 1997). Habitat associations appear to be similar between the native and non-native range, and the habitats occupied in the Iberian Peninsula enable common waxbills to reach population densities comparable to those in the native areas; based on data from Sullivan et al. (2015a) common waxbills reach densities of up to 30 individuals per ha (mean 2.1 individuals per ha), while Sanz-Aguilar et al. (2014) report ringing over 100 individuals at a single location, cf. native range population density of 2.3 individuals per ha in Swaziland (Monadjem 2002). We document selection of *A. donax*, a non-native reed, for shelter and to a lesser extent feeding. Such positive interactions between non-native species have been widely documented (e.g. Adams et al. 2003), although as *A. donax* does not occur in the common waxbill’s native range this association must have developed in the Iberian Peninsula.

## Conclusion

The spread of non-native species along multiple expansion axes provides an opportunity to disentangle the effect of climate and residence time on habitat specificity. We found that changes in the prevalence of common waxbills between the range core and range margin are likely to be driven by processes relating to residence time rather than by marginal climatic conditions, contrasting with results of previous studies of spatial variability in habitat associations of range expanding species (e.g. Oliver et al. 2009). Some changes in habitat associations were evident, with greater association of common waxbills with rivers and areas with intermediate amounts of emergent vegetation in the range margin. However, other changes in habitat associations with residence time were minor, and overall they did not translate into changes in associations with land-cover classes. These small violations of the assumption of stationarity in environmental associations mean that while species distribution models assuming stationarity are likely to be able to predict the spread of common waxbills across the Iberian Peninsula, they could be refined by incorporating changes in habitat associations with residence time. Further examples are needed in order to establish how generalizable results of this and previous studies are, with the spread of non-native species along multiple expansion axes providing a promising study system.

## Electronic supplementary material

Below is the link to the electronic supplementary material.

Supplementary material 1 (DOCX 29 kb)
