# Supplementary material for: Changes in habitat associations during range expansion: disentangling the effects of climate and residence time
Source: Biol Invasions. 2017 Nov 22;20(5):1147–59. doi: 10.1007/s10530-017-1616-9 (PMC6560651; doi:10.1007/s10530-017-1616-9)
Supplement: Supplementary file 1 — Supplementary material 1 (DOCX 29 kb) [file 10530_2017_1616_MOESM1_ESM.docx]

**Supporting materials for Sullivan & Franco “Changes in habitat associations during range expansion: disentangling the effects of climate and residence time”**

**Appendix S1.** Quantifying micro-habitat associations.

Micro-habitat associations of common waxbills were quantified using scan samples monitoring habitat use in a 180m by 180m square from a central transect along which the observer was allowed to move. This sampling square was divided into 36 30m by 30m sub-squares in which we recorded the percentage cover of each micro-habitat, allowing the configuration of micro-habitats in each sampling square to be recorded. Scan samples were performed every ten minutes, and the activity and micro-habitat association of common waxbills were recorded. Typically three scan samples were performed consecutively.

We calculated the effective area surveyed by imagining each sub-square as a three dimensional shape, where the height corresponds to detectability. If every bird was detected (i.e. detectability=1), then the volume (*V*) of each shape = 1x30x30 = 900. This can alternatively be written as equation 1, where *f(x)* is the detection function, *a* is the distance of the nearest side of the sub-square to the observer, and *b* is the distance of the furthest side of the sub-square to the observer.

$V=\int_{a}^{b} f\left( x \right) dx\times30$ Equation 1.

The effective area of a habitat in each sub-square can thus be calculated by multiplying the percentage cover of the habitat in the sub-square by *V*. The effective proportion of each habitat can then be calculated by dividing the summed effective area of each habitat by the total effective surveyed area (i.e. the sum of *V* across sub-squares).

Habitat use for a given activity at each focal watch location was calculated as the number of scan samples where common waxbills were recorded doing activity *a* in habitat *h*. Expected use of habitat *h* for activity *a* was calculated by multiplying the total number of observations of common waxbills doing activity *a* at a site by the availability of habitat *h* at that location. This gives the expected use of each habitat if common waxbills randomly selected habitat according to its availability.

Jacobs index was used to quantify habitat selection. We did not calculate this at focal watch location level due to the small number of observations at each location. Instead, we summed both observed and expected microhabitat use across focal watch locations to obtain a single value of observed (*O*) and expected (*E*) habitat use using all available data. Jacobs index was then calculated as

*J_h,a_* = (*O_h,a_* – *E*_h,a_)/ (*O_h,a_* + *E*_h,a_- 2.*O_h,a_*.*E*_h,a_) Equation 2.

We tested whether common waxbills used a habitat statistically significantly more or less than expected by calculating Bonferoni 95 % confidence intervals around the observed proportion of observations of an activity in each habitat, and assessing whether these overlapped with the expected proportion of observations of an activity in each habitat.

To examine whether microhabitat selection varied with residence time, we split our data into two sets, locations colonised by 1990, and locations colonised after 1990, and repeated the above calculation of Jacobs index. We did not further subdivide our data by residence time, due to the small number of observations in each residence time strata.

**Table S1.** Relative performance of models of common waxbill detectability.

| Model | AIC |
| --- | --- |
| EMERGE+TREE+GROUP | 2038.6 |
| **TREE+GROUP** | **2036.6** |
| EMERGE+GROUP | 2040.8 |
| EMERGE+TREE | 2041.4 |
| EMERGE | 2053 |
| TREE | 2049.7 |
| GROUP | 2049.3 |
| 1 | 2039.7 |

EMERGE is the proportion of emergent vegetation, TREE is the proportion of trees and bushes and GROUP is the flock size. 1 indicates a null model with no environmental covariates. The model with the lowest AIC is shown in bold.

**Table S2.** Microhabitat selection by common waxbills, divided by residence time strata, calculated using Jacobs index (*J*). Asterisks indicate that microhabitat use differs statistically significantly from expected use if each microhabitat was selected randomly (assessed by expected use of a microhabitat falling outside the 95% Bonferoni confidence intervals of observed proportional use).

|  | Feeding | | | Shelter | | |
| --- | --- | --- | --- | --- | --- | --- |
| Habitat | Observed | Expected | J | Observed | Expected | J |
|  | All data | | | | | |
| *Arundo donax* | 3 | 3.4 | -0.03 | 12 | 1.7 | 0.77* |
| Trees and bushes | 5 | 17.1 | -0.58* | 17 | 14.1 | 0.1 |
| Crops | 6 | 22.6 | -0.63* | 3 | 25.3 | -0.84* |
| Emergent vegetation | 19 | 8.9 | 0.45* | 44 | 9.9 | 0.75* |
| Forbs | 18 | 15.1 | 0.14 | 13 | 14.2 | -0.07 |
| Houses and gardens | 1 | 1.7 | -0.23 | 1 | 2.4 | -0.43 |
| Rough grass | 34 | 22 | 0.35* | 6 | 25.4 | -0.7* |
|  | Residence time ≥ 20 years | | | | | |
| *Arundo donax* | 0 | 1.3 | -1* | 11 | 0.87 | 0.87* |
| Trees and bushes | 2 | 11 | -0.73* | 13 | 0.38 | 0.38 |
| Crops | 2 | 14.5 | -0.8* | 2 | -0.81 | -0.81* |
| Emergent vegetation | 12 | 5.8 | 0.42 | 14 | 9.53 | 0.53 |
| Forbs | 13 | 8.4 | 0.28 | 5 | -0.12 | -0.12 |
| Houses and gardens | 1 | 1.6 | -0.2 | 1 | 0.18 | 0.18 |
| Rough grass | 27 | 16.1 | 0.41* | 2 | -0.8 | -0.8* |
|  | Residence time < 20 years | | | | | |
| *Arundo donax* | 3 | 2 | 0.26 | 1 | 0.8 | 0.1 |
| Trees and bushes | 3 | 6.1 | -0.34 | 4 | 7.7 | -0.35 |
| Crops | 4 | 8.2 | -0.36 | 1 | 12.4 | -0.88 |
| Emergent vegetation | 7 | 3.1 | 0.5 | 30 | 4.9 | 0.88* |
| Forbs | 5 | 6.7 | -0.12 | 8 | 8.5 | -0.02* |
| Houses and gardens | 0 | 0.1 | -1 | 0 | 1.8 | -1 |
| Rough grass | 7 | 5.9 | 0.18 | 4 | 12.8 | -0.59* |

**Table S3.** Parameter estimates of lowest AIC model of common waxbill occurrence.

|  | Estimate | SE | Z | P |
| --- | --- | --- | --- | --- |
| Intercept | -1.93 | 0.83 | -2.34 | 0.019 |
| Date | 0.02 | 0.01 | 3.17 | 0.002 |
| Emerge | 1.11 | 4.42 | 0.25 | 0.802 |
| Emerge^2^ | -10.38 | 4.64 | -2.24 | 0.025 |
| Residence.time | 18.44 | 4.40 | 4.19 | <0.001 |
| Residence.time^2^ | -12.29 | 3.75 | -3.28 | 0.001 |
| Trees | -7.38 | 2.96 | -2.49 | 0.013 |
| Trees^2^ | -0.65 | 3.17 | -0.20 | 0.838 |
| Grass | -4.59 | 3.84 | -1.20 | 0.232 |
| Grass^2^ | -11.41 | 3.85 | -2.96 | 0.003 |
| River | 0.82 | 0.46 | 1.79 | 0.073 |
| Forb | 9.02 | 3.28 | 2.76 | 0.006 |
| Forb^2^ | -9.92 | 3.12 | -3.18 | 0.001 |
| Emerge:Residence.time | 68.04 | 93.64 | 0.73 | 0.467 |
| Emerge:Residence.time^2^ | 210.20 | 100.60 | 2.09 | 0.037 |
| Emerge^2^:Residence.time | -65.49 | 74.52 | -0.88 | 0.379 |
| Emerge^2^:Residence.time^2^ | -159.90 | 76.80 | -2.08 | 0.037 |
| Trees:Residence.time | -24.17 | 58.11 | -0.42 | 0.677 |
| Trees:Residence.time^2^ | -113.70 | 55.82 | -2.04 | 0.042 |
| Trees^2^:Residence.time | -17.26 | 59.80 | -0.29 | 0.773 |
| Trees^2^:Residence.time^2^ | 185.30 | 57.42 | 3.23 | 0.001 |
| Grass:Residence.time | 111.70 | 74.16 | 1.51 | 0.132 |
| Grass:Residence.time^2^ | -51.35 | 68.23 | -0.75 | 0.452 |
| Grass^2^:Residence.time | 103.50 | 77.33 | 1.34 | 0.181 |
| Grass2:Residence.time^2^ | -23.49 | 65.40 | -0.36 | 0.719 |
| River:Residence.time | -17.79 | 9.60 | -1.85 | 0.064 |
| River:Residence.time^2^ | 5.39 | 8.30 | 0.65 | 0.516 |
| Forb:Residence.time | -142.30 | 66.16 | -2.15 | 0.031 |
| Forb:Residence.time^2^ | 107.30 | 57.69 | 1.86 | 0.063 |
| Forb^2^:Residence.time | 43.97 | 61.69 | 0.71 | 0.476 |
| Forb^2^:Residence.time^2^ | -13.17 | 59.99 | -0.22 | 0.826 |


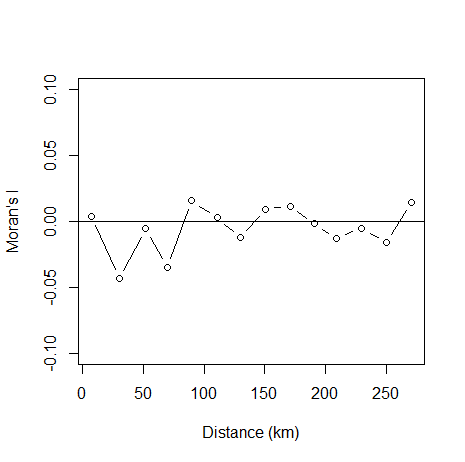


**Figure S1.** Relationship between residual spatial autocorrelation (residuals from best performing model) and distance between point count locations. No distance class exhibited statistically significant residual spatial autocorrelation, assessed following 500 permutations.
